# Enhanced Gas Sensing Properties of Spin-coated Na-doped ZnO Nanostructured Films

**DOI:** 10.1038/srep41716

**Published:** 2017-02-01

**Authors:** Mohamed A. Basyooni, Mohamed Shaban, Adel M. El Sayed

**Affiliations:** 1 Nanophotonics and Applications (NPA) Lab, Department of Physics, Faculty of Science, Beni- Suef University, Beni- Suef 62514, Egypt; 2 Space Research Lab, Solar and Space Research Department, National Research Institute of Astronomy and Geophysics (NRIAG), Helwan, Cairo, Egypt; 3 Department of Physics, Faculty of Science, Fayoum University, Fayoum 63514, Egypt

## Abstract

In this report, the structures, morphologies, optical, electrical and gas sensing properties of ZnO and ZnO: Na spin-coated films are studied. X-ray diffraction (XRD) results reveal that the films are of a single phase wurtzite ZnO with a preferential orientation along (002) direction parallel to *c*-axis. Na doping reduces the crystalline quality of the films. The plane surface of ZnO film turned to be wrinkle net-work structure after doping. The reflectance and the optical band gap of the ZnO film decreased after Na doping. The wrinkle net-work nanostructured Na-doped film shows an unusually sensitivity, 81.9% @ 50 sccm, for CO_2_ gas at room temperature compared to 1.0% for the pure ZnO film. The signals to noise ratio (SNR) and detection limit of Na-doped ZnO sensor are 0.24 and 0.42 sccm, respectively. These enhanced sensing properties are ascribed to high surface-to-volume ratio, hoping effect, and the increase of O- vacancies density according to Kroger VinK effect. The response time increased from 179 to 240 s by the incorporation of Na atoms @50 sccm. This response time increased as the CO_2_ concentration increased. The recovery time is increased from 122 to 472 s by the incorporation of Na atoms @50 sccm.

Carbon dioxide (CO_2_) sensors play an important role in monitoring and controlling indoor air quality. Sensing of CO_2_ using inexpensive, miniaturized, highly sensitive sensors that work at room temperature is of great interest for environmental, agricultural applications, Martian atmosphere, and consumer applications^1^. Considering the tremendous impact of CO_2_ emissions on the global warming, monitoring of CO_2_ has a vital importance in the field of gas sensors. CO_2_ sensors are also critical in the food processing industry to maintaining the freshness and shelf life of food, breath analyzers in healthcare, environmental incubators in biotechnology and petrochemical plants in the industry^1^. Also, there is a highly significant need for CO_2_ sensors for space and commercial applications including low-false-alarm fire detection inside the spacecraft which detects chemical species indicative of a fire (CO_2_ and CO). The Martian atmosphere is primarily CO_2_ with a balance of nitrogen, argon and trace species^2^. Nevertheless, there are only limited studies in the CO_2_ sensing materials which able to work at room temperature, due to the high stable chemical properties of CO_2_ gas. Chemical CO_2_ gas sensors based on metal oxides exhibit low energy consumption, simplicity, and small size in comparison with spectroscopic sensors. Hence, much attention has been paid to find or design new CO2 sensitive compounds that able to work at room temperature and could ensure specificity, ambient conditions operations, high sensitivity, fast and reversible response^3^.

Transparent conducting oxide (TCO) films based on zinc oxide (ZnO) are one of the most credible candidates for spintronics, light emitting and laser diodes, solar energy conversion, storage device, paint coatings, and antibiotic. This is owing to that ZnO has a broadband gap (3.3 eV), a large exciton binding energy (60 meV), its abundance, eco-friendliness and other distinctive properties^4^,^5^. ZnO films have thermal diffusivity in the range (4.35–5.03) × 10^−2^ cm^2^/s and electrical resistivity varying from 10^−4^ to 10^12^ Ω.cm^6^. The as-grown ZnO exhibits n-type conductivity due to native defects such as Zn interstitial (Zn_i_) and O-vacancy or due to the unintentionally incorporated hydrogen during crystal growth^7^. ZnO is one of the most widely used as gas sensing materials due to its low fabrication cost and high electron mobility^8^. However, being a promising material for the gas sensor device for monitoring some gases like CO and H_2_S and CH_4_^9^, ZnO still faces many problems such as poor sensitivity, high operating temperature, and low reliability which might limit its applications for gas sensor^10^. Many approaches have been made to modify the sensing properties of ZnO films to reduce the operation temperature and achieve high sensitivity. For that, different approaches are adopted including new methods of ZnO synthesis, doping with suitable metals, and new nanostructure morphologies.

To face these drawbacks, many groups tried to use nanostructured ZnO. Ghobadifard *et al*.^11^ reported that 0.012 ppm^−1^ sensitivity, 0.5 ppm detection limit, and 150 s response time for 100 ppm CO_2_ at 300 °C could be achieved using hydrothermally prepared nanocrystals ZnO thick film on an inter-digitated alumina substrate. Kannan *et al*.^12^ demonstrated that nanostructured ZnO thin film of thickness 40 nm, deposited on glass substrates using a DC reactive magnetron sputtering technique, is a better CO_2_ sensor than thick ZnO films. The maximum sensitivity was 1.13% at 300 °C, the response and recovery times were observed at 1000 ppm to be 20 s. Pan *et al*.^13^ showed that ZnO hierarchical nanostructure-based gas sensor was directly and locally grown on a single silicon chip for sensitive detection of NO_2_. They reported RT output response of 32, response time of 72 s and recovery time of 69 s at 20 ppm NO_2_.

Also, many other working groups tried to overcome these problems by doping thin films. Samarasekarap *et al*.^14^ reported sputtered ZnO thin films as a CO_2_ gas sensor; the sensitivity was measured to be 2.17 at 100 °C and the response and recovery times were 5 s and 10 min, respectively. Nemade *et al*.^15^ reported that ZnO thin film prepared by a screen-printing method on a glass substrate with a sensitivity of ~ 0.9 for 200 ppm towards CO_2_ at RT. Xiao *et al*.^16^ have examined Co-doped ZnO sensors with different Co contents to ethanol, and they demonstrated that the 3 mol% Co-doped ZnO sample showed the highest response value to 100 ppm ethanol at 350 °C, which was five folds greater than that of the pure ZnO sample. Rai *et al*.^17^ have shown that CuO-nanoparticles surface functionalization leads to a four times increase in ZnO sensitivity to 1000 ppm of CO. It has been observed that the Cu-doped ZnO thick films are more sensitive to Liquefied Petroleum Gases (LPG) than other tested gases viz: NH_3_, CO_2_, H_2_S, Ethanol and NO_2_^18^. The ZnO thick films doped with 5 wt. % Cu has shown higher sensitivity to LPG than other doping concentrations. These films have shown 87.80% sensitivity to LPG at 300 °C operating temperature and 22.22% for CO_2_ gas. Patil *et al*.^19^ prepared a thick film of pure and Al-doped ZnO on alumina substrates using a screen printing technique. He showed that the Al-doped films illustrated significant sensitivity to CO_2_ gas than pure ZnO film at 250 °C. The sensitivity of pure ZnO film to CO_2_ was found to be 12.1% at 300 °C. Pure ZnO is notably less sensitive than doped ZnO. The sensitivity of 10 wt. % Al doped ZnO film was observed as 87.3% at 250 °C. The response time was ~ 25 s to 1000 ppm of CO_2_ while the recovery was ~ 110 s. Herrán *et al*.^20^ used photoactivated BaTiO_3_–CuO thin film for CO_2_ detection at RT, which showed a logarithmic sensitivity relation at CO_2_ concentration from 500 to 5000 ppm and a response time of around 2 min. Hoefer *et al*.^21^ developed a sensor film based on SnO_2_ which was most responsive in the range 2000–5000 ppm of CO_2,_ but out of this range, the response is very weak.

Sodium (Na) is a suitable acceptor from the group I. Na delivers a high hole concentration up to 3 × 10^18^ cm^−3^ and possesses a relatively shallow substitutional level (Na_Zn_: 0.17 eV)^22^. Hence, Na is an excellent substitute for Zn^23^,^24^,^25^. Na can also enter the ZnO lattice interstitially in combination with a neighboring oxygen vacancy^26^,^27^. Na-doped ZnO structures are a candidate for self-cleaning coatings^28^. Also, they were reported as good blue emission materials with an excellent photocatalytic activity for organic pollutants in water^29^. Various Na-doped ZnO nanostructures such as microwires^25^, nanowires^27^,^29^, and thin films^23^,^24^,^30^,^31^,^32^ have been prepared by chemical vapor deposition (CVD)^25^, thermal decomposition^29^, RF-sputtering^27^, sol-gel^28^,^30^,^31^,^32^, pulsed laser deposition (PLD)^23^,^33^, and metal–organic chemical vapor deposition (MOCVD)^24^. Among these techniques, the sol–gel method offers a controllable and low-cost way for the preparation of mixed oxide systems based on ZnO. The advantages of the sol–gel approach includes homogeneity of the obtained thin films, excellent control of the stoichiometry, ease of compositional modification, large area substrate coating and the ability to scale up to industrial fabrication^34^. Based on the above survey, no complete report on gas sensing properties of Na-doped ZnO spin coated films at high or RT. Thus, this work is devoted to studying the influence of 2.5% Na doping on the morphological, structural and optical properties as well as the films’ sensitivity towards CO_2_ and N_2_ gases of sol-gel spin coated ZnO films.

## Results and Discussion

### XRD analysis

**Figure 1:**
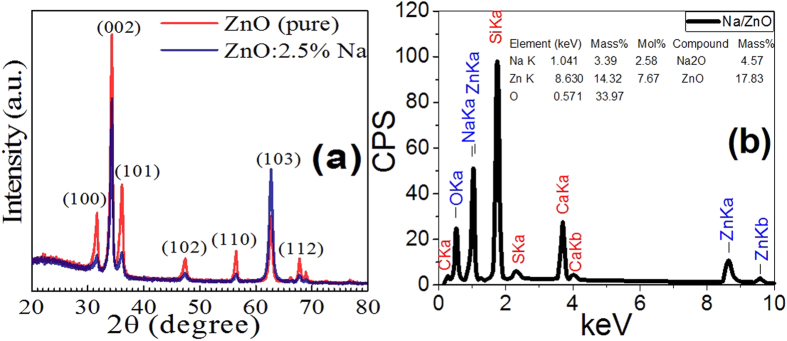
(**a**) XRD spectra of pure and 2.5% Na-doped ZnO films and (**b**) EDX spectrum of 2.5% Na-doped ZnO film.

Figure 1(a) shows the XRD spectra of ZnO (pure) and Zn_0.975_Na_0.025_O films. These films show polycrystalline nature. All the peaks are ascribed to the hexagonal wurtzite ZnO (JCPDS-89-0510)^35^. No peaks related to metallic Na or Na compounds are observed. So, Na atoms may be substituted Zn atoms or incorporated into interstitial sites in the ZnO lattice^26^. This implies that the precursors have been completely converted into ZnO phase, and Na doping did not alter the hexagonal structure of the ZnO lattice. No shifts are detected for the characteristic peaks although the differences between the ionic radii of the host and the dopant, *r*_Zn_ = 0.74 Å and *r*_Na_ = 0.95 Å^31^,^36^. However, introducing Na into ZnO lattice reduces the peak’s sharpness except for the (103) reflection peak. This may be ascribed to the significant difference in the ionic radii between the host and the dopant which introduces lattice defects and affect the film crystallinity. Li *et al*. showed a marginal shift of the characteristic peaks ((100), (002) and (101)) of chemically prepared ZnO nanoparticles toward lower diffraction angle after doping with Gd, Er, and Li^37^. Raza *et al*.^38^ detected no impurity phases for 1.2% Ce or La - doped ZnO nanoparticle prepared by sol-gel technique. Li *et al*. observed a diffraction peaks shift towards lower 2*θ* values accompanied with the existence of a secondary phase ZnSrO_2_ in the hydrothermally prepared 0.3% Sr- doped ZnO^39^. The present results refer that the sol-gel spin coating is an efficient method to produce ZnO films free from impurity phases. Despite the fact that, there are no secondary phases recognized by XRD investigation, the presence of secondary phases can’t be altogether prohibited because of the limitation of this characterization method^40^.

The texture coefficient (*TC*) of a particular plane (*hkl*) is calculated utilizing the following equation^39^:


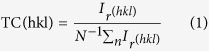


where *n* is the number of diffraction peaks, *I*_*r*_*(hkl)* is the ratio between the measured relative intensity of a plane *(hkl)* to its standard intensity taken from the *JCPDS* data, and *N* is the reflection number. *TC*s values for the first three characteristic peaks ((100), (002) and (101)) are calculated and presented displayed in Table 1. The ZnO films exhibit upgraded intensities relating to (002) peak when contrasted with (100) and (101) peaks, which shows a preferential orientation along the *c*-axis. Comparable results were accounted for the hydrothermally produced Na-doped ZnO films and nanowires^27^,^30^. This may be ascribed to the values of surface free energy (SFE) of (002), (110) and (100) plans. The three lowest densities of the SFE are 9.9 for (002), 12.3 for (110) and 20.9 eV/nm^2^ for (100) plan^26^. TC (002) of pure ZnO is greater than that of 2.5% Na - doped ZnO film. Similar results have been reported for Na-doped ZnO films and Er, La and Yb-doped ZnO nanocrystals^28^,^35^,^41^. This highly preferred orientation along *c*-axis is important for piezoelectric applications including transducers devices and ultrasonic oscillators^5^.

The crystallite size (*D*) of ZnO (pure) and Zn_0.975_Na_0.025_O films was determined using the well known Scherer’s formula^35^. Using the full width at half maximum intensity of the first three peaks; (100), (002) and (101), the average values of *D* are obtained and listed in Table 1. The *D* value decreases from 34.22 to ~ 19.54 nm after Na doping. This reduction in size may be ascribed to the crystallinity deterioration, the reduction in the peak’s sharpness and the increase in the full width at half maximum intensity, after doping with 2.5% Na and/or the formation of Na–O–Zn in the crystal lattice, which plays a significant role in hindering the crystal growth^37^.

The lattice constants (*a* and *c*) of the films are calculated by using the following equations^35^,^42^:


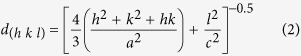


For (*h k l*) = (0 0 2) and (1 0 1), 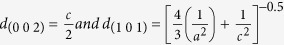, respectively. The volume, V, of the unit cell of the hexagonal ZnO is 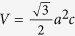. The Zn–O bond length (*L*) is given by equation (3)^4^,^40^:


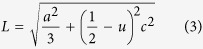


The calculated values of *a,c, V,u*, and *L* are listed in Table 1. The *V* values of pure ZnO film is 47.779 Å^3^ and increased to 48.175 Å^3^ at 2.5% Na. The same behavior can be observed for the Zn-O bond length; *L* increases from 1.980 Å to 1.986 Å after Na doping. These results may indicate that Na atoms incorporate inside ZnO lattice as substitutional atoms. Similar results were reported for Cd-doped ZnO prepared by PLD^43^.

To confirm the existence of Na element, Fig. 1(b) shows the energy dispersive X-ray (EDX) spectrum of 2.5% Na-doped ZnO film. This EDX spectrum clearly confirms the presence of Zn O, and Na peaks. There are three peaks relevant to Zn at around 1, 8.6, and 9.6 keV and peak at 1.041 keV for Na element. The quantitative analysis, inserted table in Fig. 1(b), shows 2.58 mol % for Na element. The C, Si, S, and Ca signals are detected from the glass substrate in the EDX pattern because EDX has a larger interaction volume (≥1 *μ*m at accelerating voltage ≥11 keV) than the thickness of the ZnO film. This means that the substitution for Zn in the ZnO lattice or incorporation of Na into interstitial sites in the ZnO lattice could occur regardless of the differences in the atomic radii of Na and Zn.

### Films Morphology

**Figure 2:**
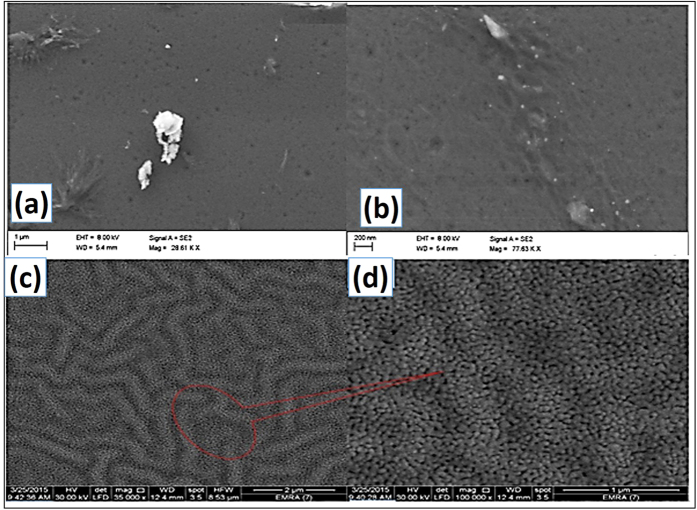
FE-SEM images at two different magnifications for (**a,b**) pure ZnO and (**c,d**) for 2.5% Na-doped film.

Figure 2 illustrates the surface microstructure images (FE-SEM) of ZnO (pure) and Zn_0.975_Na_0.025_O films. The flat ZnO surface turned to a wrinkle network structure that consists of dense grains from agglomerated nanoparticles with narrow particle size distribution. These nanoparticles were self-assembled to produce nanoporous wrinkle network structure with pores of diameters <30 nm. The average value of nanoparticle diameter is 39 nm, which is two times the XRD crystallite size (Table 1). This indicates that the grain size or nanoparticle size that measured by FE-SEM might be comprised of more than one crystallite which measured by the XRD. Sharma *et al*. reported similar results for ZnO structures doped with Sc^44^. Finally, the particles constituting these films are of narrow size distribution, and the number of particles per unit area is very high. So the Na-doped film is appropriate for different nanotechnological applications such as sensors and photocatalysts.

### Optical Properties of the films

**Figure 3:**
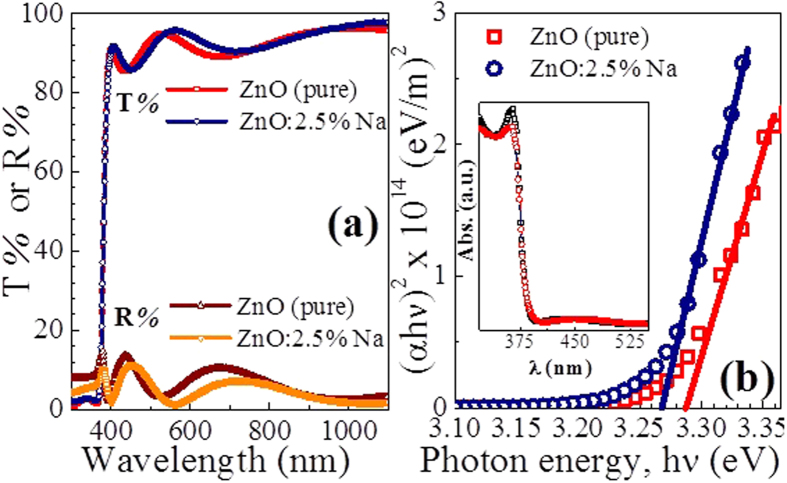
(**a**) Transmission (*T*) and reflectivity (*R*) spectra and (**b**) the plots of (*αhν*)^2^ & *hν* to determine *E*_*g*_ values for pure and 2.5% Na-doped ZnO films. The inset shows the absorption spectrum of the films.

The optical transmittance (T) and reflectivity (R) spectra of the pure and 2.5% Na-doped ZnO films in the 300–1100 nm domain were corrected for the substrates effects^45^ and are depicted in Fig. 3(a). The un-doped and Na-doped ZnO films showed a high transmittance of up to 97% in the visible region. Additionally, these films showed sharp fundamental absorption band edges near to *λ* = 380 nm. This high transmittance is attributed to small scattering effects resulting from the structural homogeneity of the films and the apparent high crystallinity (Fig. 1). Similar to that were reported for Sn-doped ZnO films grown by spray pyrolysis^6^. The T values are slightly increased after doping with Na. Also, R values of undoped films are less than 15%, and the R of the Na-doped film is smaller than that of the undoped one, as shown in Fig. 3(a). The T and R plots of the two films showed interference patterns at various *λ*. This phenomenon was also reported in the transmittance spectra of (Mg, Ga) and (Al, Ga) - co-doped ZnO films that fabricated by dip coating technique^46^,^47^. Continuously oscillating maxima and minima at different wavelengths suggest the optical homogeneity of deposited thin films. Moreover, within the IR region, the *R* values are almost below 5%, this indicates that the films seem exempted of free charges.

From the transmittance spectra, the absorption coefficient and the extinction coefficient (*α* and *k*) of the undoped and Na-doped ZnO films were calculated according to the relations^35^,^45^:


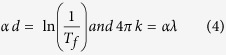


where *d* is the film thickness. For the direct allowed transition, the relationship between *α* and photon energy (*hν)* is given by^35^:


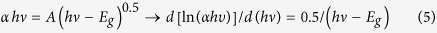


where *A* is a constant, *h* is the Planck’s constant, and *E*_*g*_ is the optical band gap. From Eq. (5), at *E*_*g*_ = *hν,* the plot of d[ln(*αhν*)]/d(*hν*) versus *hv* will result in a discontinuity. Then, the values of *E*_*g*_ can be obtained by extrapolating the linear part of the plots of (*αhν*)^2^ vs. *hν* to *α* = 0, as shown in Fig. 3(b). The corresponding *E*_*g*_ values are given in Table 1. As seen, the *E*_*g*_ value decreases from 3.26 eV, for pure ZnO, to 3.23 eV after Na doping. This *E*_*g*_ shift is ascribed to the change of lattice constant of ZnO crystal and subsequently the lattice distortion due to the generated defects after Na doping and the introduction of doping levels/impurity band in ZnO lattice. Similar results were reported for Sr-doped ZnO^39^ and the sol-gel prepared Ce- and La-doped ZnO nanoparticles^32^,^38^. Thus, the doping results in the rise of additional band tail states, leading to shrinkage of the band gap. In contrast, Nd doping was found to increase the *E*_*g*_ values of the ZnO spin coated films^48^.

The band gap tail in the valence and conduction bands (Urbach energy, *E*_*U*_) is attributed to the disorder in the material^49^. *E*_*U*_ can be calculated from the slope of the linear fitting of the plot of *ln(α*) vs. *hν* according to the relations 
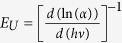
49. The obtained *E*_*U*_ values are 62.15 and 67.96 meV for ZnO and Na-doped ZnO films, respectively. The increase in the *E*_*U*_value confirms the XRD measurements, where the crystalline plane of pure ZnO is more oriented along (002) direction than Na-doped ZnO film.

**Figure 4:**
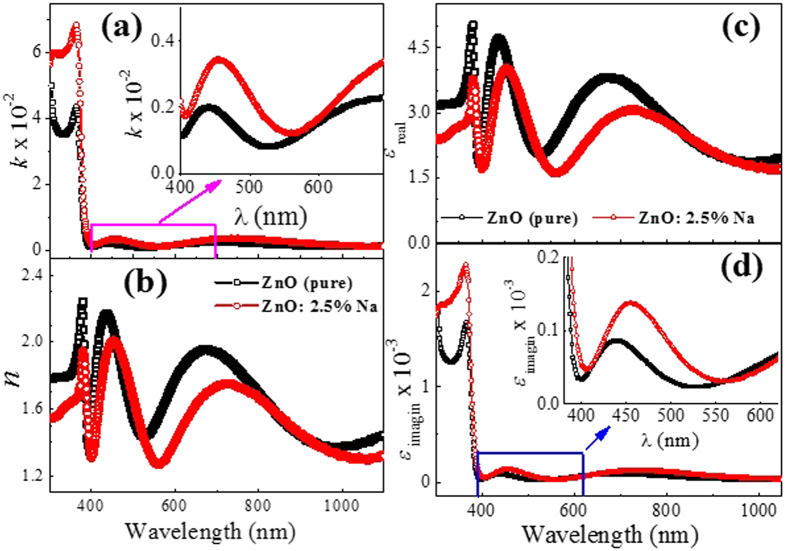
(**a**) The dependence of the absorption index, *k*, (**b**) the refractive index, *n*, (**c**) the real part and (**d**) the imaginary part of the dielectric constant of the ZnO and ZnO: 2.5%Na films with wavelength.

The dependence of the absorption index *k*, (*k* = *αλ/4π*), on the wavelength, is shown in Fig. 4(a). In the UV and visible regions (as seen from the inset of this figure), the *k* values of ZnO: 2.5% Na is higher than that of pure ZnO. This means that the doping of ZnO with Na enhances its uses in blocking the UV radiations. Utilizing the corrected values of the reflectance, *R*, the refractive index, *n,* of the films was determined according to the relation^35^,^49^; 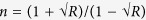. The dependence of *n* values on λ are shown in Fig. 4(b) which shows the oscillatory behavior of the refractive index. For pure ZnO, the maximum (*n*_*max*_) and minimum (*n*_*min*_) values are between 2.2 and 1.37. For Na-doped film, these values are decreased to be between 2.0 and 1.25. This oscillatory behavior of *n* values was also reported for Mg-doped SnO_2_ films^50^.

The real and imaginary parts of the dielectric constants; *ε*_*real*_ and *ε*_*imagin*_ given by; 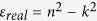 *and ε*_*imagin*_=2*n k (where ω* is the angular frequency and *ε*_*o*_ is the free space dielectric constant), are shown in Fig. 4(c,d). The values of *ε*_*real*_ for pure ZnO are oscillating between 2.0 and 5.0, whereas, these values are oscillating between 2.5 and 4.0 for ZnO: 2.5%Na. Also, it is seen that the *ε*_*real*_ values are more than 10^3^ greater than that of *ε*_*imagin*_. Moreover, *ε*_*imagin*_ values are nearly constant in the visible region, but increasing sharply on approaching the UV region due to the strong interaction between the highly energetic photons and the charge carriers of the material.

### I-V characteristics

**Figure 5:**
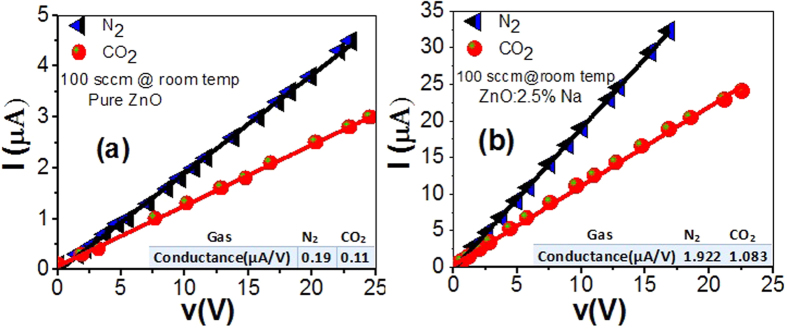
I–V characteristics of (**a**) pure and (**b**) 2.5% Na – doped ZnO sensors to CO_2_ and N_2_.

I-V measurements provide information about the contact type and the particle-to-particle and electrode-particle contact formation. The CO_2_ detection ability of the ZnO and Na-doped ZnO nanostructured films that annealed at 500 °C were tested in a homemade gas chamber under nitrogen and the CO_2_ environment. The characteristic I-V curves of both samples in N_2_ and CO_2_ environment of 100 sccm concentration were measured for 5 min at RT and represented in Fig. 5. This figure clearly shows linear Ohmic behavior for both films. When the environment was switched from CO_2_ to N_2_, the rate of the follow of current at the same voltage increased to ~two folds. This may be ascribed to the oxidizing nature of CO_2_ gas which leads to the increase of the resistance of the n-type material. However, the nature of N_2_ gas leads to the decrease of the Ohmic resistance^51^.

By incorporating of Na atoms and the change of ZnO flat surface to wrinkle network nanostructure, the current density and conductance are increased to greater than ten folds. This may be ascribed to many reasons as follows. First, in Na-doped ZnO, Na acting as an acceptor when substituting for Zn and as a donor when occupying interstitial sites (amphoteric behavior)^52^. In addition to behaving as deep acceptors, Meyer *et al*. reported that Na, incorporated either by diffusion or during thin-film growth, can also result in (relatively) shallow acceptors with binding energies around 300 meV, with a weak electron–phonon coupling and a high conductivity^53^. This agrees well with the decrease of the band gap of ZnO by the incorporation of the Na atoms as shown in Table 1. Secondly, the gas molecules adsorbed in the pores of the wrinkle network structure induce band bending to the associated nanoparticles such that the contact potential can be modified depending on the nature of the gas molecules. The extent of band bending increases the conducting width and decreases the potential barrier of the contacts between the nanoparticles, therefore causes an increase in the conductivity. Thirdly, the high surface to volume ratio becomes a dominant factor to control the resistance of the nanostructured film. After exposure, gas molecules are adsorbed on the surface and pores of the wrinkle network film by sharing donor electrons from the nanoparticles. The inserted tables in Fig. 5 show the values of the conductance in N_2_ and CO_2_ environment for un-doped and Na-doped ZnO films.

### Dynamic Response Curve Analysis

**Figure 6:**
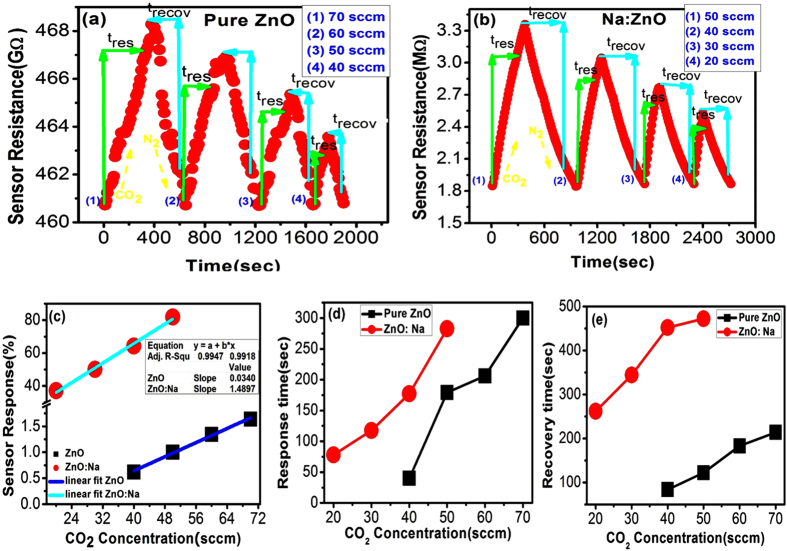
The dynamic response of (**a**) pure ZnO and (**b**) 2.5% Na- doped ZnO sensors with the detection time; (**c**) The sensor response % as a function of CO_2_ concentration in sccm unit; and (**d**) response time (t _res_) and (**e**) recovery time (t _recov_) of pure and 2.5% Na- doped ZnO vs. CO_2_ concentration in sccm unit.

The dynamic response behavior of the pure and 2.5% Na-doped ZnO sensors are shown in Fig. 5. These curves represent the adsorption-desorption reaction of the gas interaction process on the thin film surface. The detection, response and recovery time of the target gases are important indices of the adsorption-desorption kinetic process and are crucial parameters for designing sensors for the desired application. When the CO_2_ gas was introduced, the resistance of the film, R_s_, is increased with the detection time and reached a steady state. At this point, CO_2_ gas flow was turned off, and N_2_ gas was introduced into the test chamber. Figure 6(a) shows the dynamic response of pure ZnO towards CO_2_ of concentrations 40, 50, 60, and 70 sccm for 5 minutes in an inert environment at RT. Figure 6(b) shows the same behavior for 2.5% Na-doped ZnO for CO_2_ concentrations of 20, 30, 40, and 50 sccm at the same conditions. The lowest detected concentration using our experimental set-up is 40 sccm for ZnO and less than 20 sccm for Na-doped ZnO. It can be observed for both cases that upon exposure to CO_2_, the resistance of the film increases which confirms its n-type semiconducting behavior^54^. The sensor resistance value was found to decrease rapidly and reach the baseline value and in turn revealed the excellent recovery characteristics of the sensor.

The sensor response (sensitivity%) was calculated using^55^:


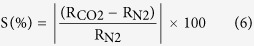


where R_N2_, R_CO2_ are the sensor resistances in the presence of N_2_ and CO_2_, respectively. The sensor response %, sensor sensitivity (S %), as a function of CO_2_ concentration, was calculated using Eq. (6) and illustrated in Fig. 6(c) and Table 2. The sensitivity of pure ZnO is increased linearly from 0.6 to 1.7% as CO_2_ concentration increased from 40 to 70 sccm at RT. Na-doped ZnO film shows very high sensitivity compared to that of pure ZnO film. The sensitivity of Na-doped ZnO film is linearly increased from 37% to 81.9% as the CO_2_ concentration increased from 20 to 50 sccm at RT. From the linear fitting of the experimental data, the rate of increase of sensitivity with respect to CO_2_ concentration is 0.034/sccm for ZnO and 1.49/sccm for Na-doped ZnO. This indicates resolution enhancement of almost 44 fold by incorporating 2.5% Na. The correlation coefficient R^2^ = 0.992 which represents the high quality of the curve fit^56^.

The detection limit of the sensor was calculated to form the sensor’s dynamic curves as the following. Signal to noise ratio (SNR) is calculated from Eq. (7)^56^:


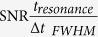


where *t*_*resonance*_ is the resonance response time and 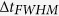 is the full width at half maximum of the response peak. The rms noise is given by Eq. (8)^56^:


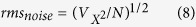


where *N* is the number of data points used in the curve fitting and 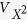 is given by


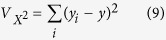


where y_i_ is the measured data point and y is the corresponding value calculated from the curve-fitting equation. The values of SNR are calculated using Eq. (7) and listed in Table 3 for 2.5% Na- doped ZnO film, Fig. 6(b). The average noise level is 0.241362 for the CO_2_ sensor. According to the IUPAC definition^57^, the detection limit can be extrapolated from the linear calibration curve when the signal equals three times the noise (DL (sccm) = 3 *rms*/slope). The CO_2_ detection limit is calculated to be 0.420658 sccm.

The response time, t_res_, is defined as the time required to achieve 90% of the total resistance change when CO_2_ is introduced into the chamber. The recovery time, t_recov_, is the time that required to reach 90% of the total resistance change when CO_2_ is turned off, and N_2_ is re-introduced into the chamber. The calculated response and recovery time from Fig. 6(a,b) for both sensors at different CO_2_ gas concentrations are depicted in Fig. 6 (d) and (e) and Table 2. The lower the durations of detection, response, and recovery, the better are the sensors. The average response and recovery times for the sensors were observed to increase with increasing CO_2_ concentration. At the same concentration, Na-doped ZnO sensor shows higher response and recovery time than pure ZnO sensor.

### Sensing mechanism of ZnO and Na-doped ZnO at RT

Here we discuss the sensing mechanism of ZnO and Na-doped ZnO films as CO_2_ sensors. ZnO is n-type semiconductor for its dominant electrons contributed by the oxygen vacancy and Zn interstitial because ZnO has a very rich defect chemistry^58^.

The surface adsorbed CO_2_ gas (oxidizing agent) captures electrons from ZnO material and led to increasing the overall resistance of the ZnO film^59^. The ZnO film exhibits poor response due to its limited surface to volume ratio. The increase of surface to volume ratio (e.g. nanoporous materials) makes it possible to lower the required operating temperature, reduce the power needed for heating, and enhance the sensor output response. At the nanoscale, any change in the surface depletion region can cause a higher response of the ZnO nanostructure. Pan *et al*.^60^ studied the high response of the ZnO nanowires at high temperature. However, this improvement is still not enough to make the nanowire work properly at RT of ~25 °C^60^.

Here, the novel porous-network Na-doped ZnO nanostructured film (Fig. 2(c,d)) is proposed to extend further the response of the RT sensors for the following reasons. This film exhibits a unique morphology and conductance compared to the pure ZnO in N_2_ and the CO_2_ environment. Additionally, the small band gap is another key to enable Na-doped ZnO sensor to operate at RT. Furthermore, upon the exposure to CO_2_, the bulk resistance of the film increases more than thirty times @ 20 sccm due to the growth of grain boundary resistance, indicating that both grain boundary and intra-grain regions contribute to gas sensitivity^61^. Then, the response of ZnO towards CO_2_ rises with introducing Na.

Similar behavior was observed by Davydova *et al*., wherein the resistance of the sensor at RT was increased or decreased due to the electrolytic dissociation of the gasses in the surface layer^62^. Similarly, Gao *et al*.^63^ suggested sensing reaction enhancement at the interface between SnO_2_ and lanthanum oxide. Pure ZnO has low conductance, but the addition of Na^+1^ increases the conductance of ZnO with a change in its microstructure. ZnO has a hexagonal close-packed lattice. This structure is a relatively open structure because Zn atoms occupy half of the tetrahedral sites and all the octahedral sites are empty. Hence, there are plenty of sites for ZnO to accommodate intrinsic defects and extrinsic dopants^64^,^65^. Oxygen vacancies can be created if some Na^+1^ ions become in place of zinc site. The dissociation of oxygen vacancies followed by the adsorption of CO_2_ through the formation of NaCO_2_. The Kröger Vink notation equations are given as follow:


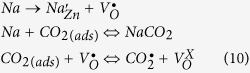


The oxygen vacancies are ionized according to the following equation:


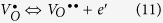


where, O = oxygen, V = vacancy, and the superscripted terms indicate charges, where a dot indicates positive charge and a prime refers negative charge.

The substitution of Zn^2+^ with Na^1+^ into ZnO crystal lattice can make more defects and vacancies. Thus, Na-ZnO contains higher concentrations of oxygen vacancies than ZnO. Then, CO_2_ adsorption and, hence, electron concentrations are increased which will enhance the sensitivity. Therefore, the CO_2_ gas was easily detected at RT and without oxygen environment, owing to the amplification effects of the combination of the percolation effect, grain boundary, and intra-grain regions.

## Conclusions

Pure and Na-doped ZnO films with a preferential *c*-axis orientation were deposited via sol-gel and spin coating method. The films’ crystallinity is deteriorated after Na incorporation. The Zn-O bond length increased from 1.980 to 1.986 Å at 2.5% Na. The volume of the unit cell of the doped films is larger than that of the undoped film. The surface of plane ZnO film is turned to wrinkle network with granular structure after Na doping. Un-doped and Na-doped films show optical homogeneity with low reflectivity and high transparency, 85–97%, in most of the studied range of the spectra. The reflectance and the optical band gap of the ZnO film decreased after Na doping. The wrinkle net-work nanostructured 2.5% Na- doped ZnO film shows very high sensitivity, 81.9% @ 50 sccm, for CO_2_ gas compared to 1.0% for flat pure ZnO. The signal to noise ratio (SNR) was 0.24, and the detection limit was 0.42 sccm for Na-doped ZnO sensor. The enhanced sensing properties of the Na-doped film may be ascribed to its high surface-to-volume ratio, hoping effect, and the increase of O- vacancies density according to Kroger VinK effect. Also, the response time was increased from 179 s to 240 sec and the recovery time was increased from 122 to 472 by the incorporation of Na atoms @50 sccm - CO_2_ concentration. The response and recovery times were increased as the CO_2_ concentration increased. These films are also suitable for use as transparent electrodes in the IR optoelectronic devices.

## Experimental details

### Materials

Zinc acetate [Zn(CH_3_COO)_2_.2H_2_O, *M*_*W*_ = 219.49, Panreac] and Sodium acetate [CH_3_COONa.3H_2_O, *M*_*W*_ = 136.08, Sigma-Aldrich] have been used as a source and dopant material, respectively, to fabricate ZnO and Na-doped ZnO thin films. 2-Methoxyethanol (2ME) [C_3_H_8_O_2_, M_W_ = 76.1] and Ethanolamine (MEA) [C_2_H_7_NO, *M*_*W*_ = 61.08, Scharlab S.L., Spain] were used as a solvent and stabilizing agent, respectively.

### Films preparation

Zinc acetate was dissolved in 7 ml of 2ME to prepare 0.5 M solution, and 0.5 M of MEA was then added to the solution. The Na additive was controlled to obtain ZnO (pure) and Zn_0.975_Na_0.025_O. Similar steps were described in our previous work^66^. The prepared mixtures were magnetically stirred at 60 °C for 2 h to obtain a clear homogeneous solution. The solution was aged for 24 h before film deposition. Glass substrates of thickness ~1.3 mm were cleaned by sonication in acetone (C_3_H_6_O), methanol(CH_3_OH), and deionized (DI) water for 10 min each. Then, the glass substrates were dried by an air gun. To remove any residual moisture, the substrates were baked at 100 °C for 20 min. The spin coating of the films on the glass substrates was carried for 30 s at 2000 rpm. Then, the coated films were dried at 180 °C for 20 min. This procedure was repeated six times for ZnO (pure) and Zn_0.975_Na_0.025_O films. After that, the as-deposited films were annealed for 120 min at 500 °C in an air furnace. Finally, the films were cooled naturally to room temperature (RT).

### Films Characterization

The structural properties of ZnO (pure) and Zn_0.975_Na_0.025_O films were investigated by X-ray diffraction (XRD, Philips X’PertPro MRD) with a step 0.021 using Cu K_α_ radiation (λ = 1.5418 Å). Field emission- scanning electron microscopy (FE-SEM; model: ZEISS SUPRA 55 VP and ZEISS LEO, Gemini Column) was used to display the morphologies of the films. The chemical compositional analysis for Na-doped ZnO film was studied using energy dispersive X-ray (EDX; Oxford Link ISIS 300 EDX). Reflectance and transmittance spectra were measured using UV/VIS/NIR 3700 double beam Shimadzu spectrophotometer in the spectral range of 300–1000 nm at RT.

### Gas sensing system and measurements

**Figure 7:**
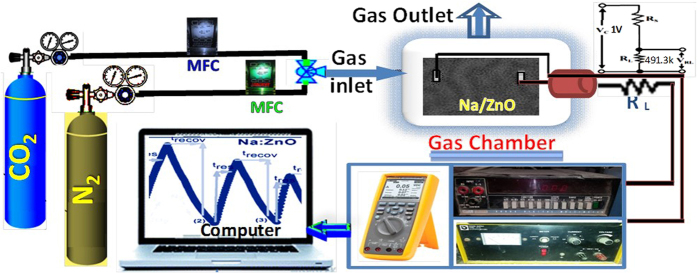
Schematic diagram of the experimental set-up of the gas sensing system.

Schematic diagram of the system used to measure the gas-sensing properties is shown in Fig. 7. This system includes the basic measuring circuit for commercially available metal-oxide gas sensors. A glass chamber of 1.0 L volume sealed with rubber O-rings on the top has been prepared for the gas sensing measurements. The top has three holes, two of them for gas inlet and outlet and the third hole to get the electrical signals. The source of the CO_2_ and N_2_ gases is a commercially available CO_2_ and N_2_ gas cylinders.

To allow a more sensitive detection of gas in our set-up, we used a load resistance R_L_. Input voltage, V_C_, of 1 V was applied from DC power supply (IMPO 11.11, 30 V/3 A). In this setup, the R_L_ is connected in series with the sensor and its value is 491.3kΩ. The output voltage, V_out_, was measured across R_L_. Nitrogen gas was flown through the sensor chamber to stabilize the sensor resistance. The sensor output signal (voltage) was recorded using Fluke Digital Multimeters 287/289 interfaced with a computer, whereas the target and carrier gases were switched on/off each cycle. It is important to note that the experimental set-up was operated at RT during the data acquisition.

The sensor was put into the glassy chamber of 1.0 L total volume. Conducting silver paste was used to make Ohmic contacts on both ends to serve as the electrodes. A different volume of CO_2_ was injected into the chamber, and the sensor output voltage was measured. The sensor resistance R_S_ was calculated with a measured value of V_out_ by using the following equation:


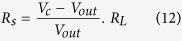


## Additional Information

**How to cite this article**: Basyooni, M. A. *et al*. Enhanced Gas Sensing Properties of Spin-coated Na-doped ZnO Nanostructured Films. *Sci. Rep.* **7**, 41716; doi: 10.1038/srep41716 (2017).

**Publisher's note:** Springer Nature remains neutral with regard to jurisdictional claims in published maps and institutional affiliations.

## Figures and Tables

**Table 1:** The XRD data for the pure and 2.5% - doped ZnO films; the texture coefficient (TC), the crystallite size (*D*), the lattice parameter (*a, c* and volume of the unit cell *V*), *u* parameter, and energy gap (*E*_*g*_).

Film	TC			*D* (nm)	Lattice parameters			*u*	*E*_*g*_ (eV)
	(100)	(002)	(101)		*a* (Å)	*c* (Å)	*V* (Å^3^)		
Pure ZnO	0.448	1.823	0.728	34.22	3.254	5.2104	47.779	0.3800	3.260
2.5% Na	0.219	2.443	0.327	19.54	3.2676	5.2099	48.175	0.3811	3.230

**Table 2:** Values of sensor response (sensitivity %), response time, and recovery time *vs*.CO_2_ concentration.

Film Type	Concentration (sccm)	sensor response (% per sccm)	response time, t_res_ (sec)	Recovery time, t_recov_ (sec)
Pure ZnO	70	1.6	300	214
	60	1.3	206	183
	50	1.0	179	122
	40	0.6	40	84
ZnO: Na	50	81.9	282.73	472.3
	40	64.1	177.0834	452.2
	30	50.0	117.56	344.4
	20	37.0	77.95	261.9

**Table 3:** Signal to noise ratio (SNR) and detection limit (DL) for Na doped ZnO.

CO_2_ Concentration	Signal to noise ratio(SNR)	Average SNR	DL (sccm)
50	0.746		
40	0.30054	0.241362	0.420658
30	0.138219		
20	0.075829		
